# Spleen stiffness in a healthy pediatric population undergoing liver magnetic resonance elastography

**DOI:** 10.1007/s00247-024-06107-z

**Published:** 2025-01-22

**Authors:** Suraj D. Serai, Alexandra Glenn, Andrew T. Trout, Wondwossen T. Lerebo, Michael S. Gee, Geetika Khanna, Sudha A. Anupindi

**Affiliations:** 1 Department of Radiology, Children’s Hospital of Philadelphia, 3401 Civic Center Blvd, Philadelphia, PA 19104 USA; 2 Perelman School of Medicine at the University of Pennsylvania, Philadelphia, PA USA; 3 University of Cincinnati, Cincinnati, OH USA; 4 Department of Radiology, Cincinnati Children’s Hospital Medical Center, Cincinnati, OH USA; 5 Department of Radiology, Massachusetts General Hospital, Boston, MA USA; 6 Department of Radiology, Children’s Healthcare of Atlanta, Atlanta, GA USA

**Keywords:** Spleen, Elastography, Pediatric, Stiffness, Normative values

## Abstract

**Background:**

Splenic stiffness is a potential imaging marker of portal hypertension. Normative spleen stiffness values are needed to define diagnostic thresholds.

**Objective:**

To report stiffness measurements of the spleen in healthy children undergoing liver magnetic resonance (MR) elastography across MRI vendors and field strengths.

**Materials and methods:**

This was a post-hoc analysis of data collected under a prospective multicenter cross-sectional study. Volunteers aged 7–17.9 years without a known history of liver or spleen disease were recruited for a research MRI between February 2018 and October 2019. Gradient recalled echo (GRE) or spin-echo–echo-planar imaging (SE-EPI) MR elastography was performed on a total of three vendor platforms and at two field strengths (1.5 T (T) and 3 T) with standard right upper quadrant passive driver placement (frequency of 60 Hz). Two independent reviewers measured spleen stiffness, length, and volume. Descriptive statistics, independent sample *t*-tests or Mann–Whitney test, and Pearson’s or Spearman’s correlation were used.

**Results:**

From 101 study volunteers, 72 (34 female) had measurable splenic stiffness. Median age was 12 years (interquartile range [IQR], 9.9–14.9 years). Mean (± SD) spleen stiffness was 4.7 ± 0.9 kPa (IQR, 3.8–5.4 kPa) with 6.1 kPa reflecting the 95th percentile. Strong correlation was observed between reviewers (ICC = 0.89 [95%CI, 0.71–0.93; *P* < 0.001]). Male volunteers had slightly higher splenic stiffness compared to females: 4.9 ± 0.9 vs. 4.3 ± 0.8 kPa (*P* = 0.014). There was significant correlation between spleen stiffness and body mass index (*r* = 0.33 [95%CI, 0.06–0.56; *P* = 0.024]) but no other measure of patient size (*r* = 0.15–0.29). No significant difference in spleen stiffness was observed across vendors (*P* = 0.089) or field strengths (*P* = 0.236).

**Conclusion:**

MR elastography–based spleen stiffness, measured as part of a liver MR elastography acquisition, is < 6.1 kPa in a healthy pediatric population and does not vary with MRI vendor or field strength.

**Graphical Abstract:**

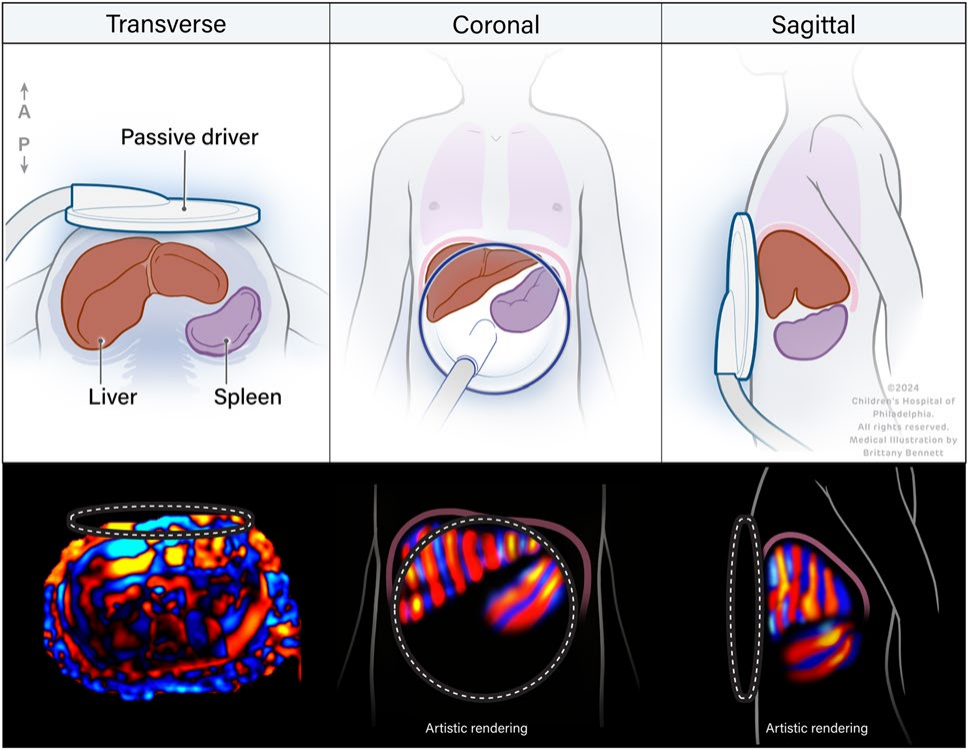

## Introduction

Magnetic resonance (MR) elastography has been established as the most accurate noninvasive technique for detecting and staging liver fibrosis [1–5]. The principle of MR elastography is to induce harmonic vibrations of acoustic-range frequencies in the tissue of interest and image the propagation of these vibrations in the tissues to estimate quantitative values of mechanical parameters [6]. Of these properties, the estimated stiffness of the organ of interest is the primary quantitative output of clinical interest. Liver MR elastography applies low-frequency (60-Hz) acoustic vibrations to induce mechanical shear waves in the liver, visualizes the shear waves by tracking tissue displacement using a modified phase-contrast sequence, and applies an inversion algorithm to generate the estimate of tissue stiffness [7].

MR elastography hardware and inversion algorithms are available on commercial scanners manufactured by at least five major MR vendors. The current regulatory-approved implementations of MR elastography available from major MRI manufacturers utilize standardized 2-dimensional (2-D) MR elastography image acquisition and inversion algorithm-based post-processing techniques (Table 1). The initial MR elastography implementations primarily utilized gradient recalled echo (GRE)–based sequences for image acquisition. Spin-echo–echo-planar imaging (SE-EPI)–based MR elastography acquisition techniques were subsequently introduced and have since been demonstrated to reduce technical failures due to rapid T2* decay in the liver, and are now widely deployed [3, 8–10]. Importantly, good agreement between the stiffness estimates generated by the two MR elastography sequence techniques has been demonstrated [3, 8, 11, 12]. In the standard MR elastography acquisition, four axial slices are acquired across four independent breath-holds (10–16 s each) for GRE MR elastography or in a single breath-hold (~ 13–16 s) for SE-EPI MR elastography.

**Table 1:** 2-dimensional (2-D) magnetic resonance elastography (MRE) scan parameters

Parameter	2-D GRE MRE	2-D SE-EPI MRE
TR (msec)	48	1,000
TE (msec)	20	30
Matrix size	256 × 64	100 × 100
Voxel size (mm)	1.6 × 1.6	1.5 × 1.5
Slice thickness (mm)	8	6
Bandwidth (Hz/px)	399	2,380
No. of slices	4	4
No. of phases	4	4
No. of averages	1	1
MEG direction	*Z* axis (slice)	*Z* axis (slice)
MEG frequency (Hz)	60	60
EPI factor	n/a	100
Echo spacing (msec)	n/a	0.5
Acceleration factor	2	2
Scan time (min:sec)	1:00 (4 breath-holds)	0:11 (1 breath-hold)

*GRE*, gradient recalled echo; *SE-EPI*, spin-echo–echo-planar imaging; *MEG*, motion encoding gradient; *TE*, echo time; *TR*, repetition time; *n/a*, not applicable; *msec*, milliseconds; *Hz*, hertz; *px*, pixel; *min*, minutes; *sec*, seconds; *mm*, millimeters

The potential clinical role of MR elastography in assessing other organs, including the pancreas, kidney, brain, lungs, or breast, is also being explored [6, 13, 14]. These applications of MR elastography are supported by evidence of excellent reproducibility and repeatability [10, 15–17]. Alternative shear wave-based methods for assessing spleen elasticity include ultrasound-based transient elastography (TE), point shear wave elastography (pSWE), and both 2-D and 3-D shear wave elastography (SWE) [18]. As compared to ultrasound-based elastography methods, MR elastography offers superior spatial resolution, greater depth penetration, larger regions of interest in the organ of interest, and comprehensive anatomical imaging, making it more effective for detailed tissue characterization [2, 19]. Given the portal venous interconnection of the liver and spleen, and known splenic manifestations of liver disease, there has been some exploration of the contribution of measuring spleen stiffness to diagnosis of portal hypertension, staging of liver cirrhosis, or predicting esophageal varices in chronic liver disease [20–23]. Additionally, spleen stiffness values have been shown to be useful in staging liver fibrosis [24]. Combining spleen stiffness with liver stiffness may offer greater diagnostic sensitivity for predicting advanced fibrosis than assessing liver stiffness alone. To date, studies of MR elastography-based splenic stiffness in healthy individuals, which have relevance to defining diagnostic thresholds, are limited [25]. The goal of this study was to compile normative data for MR elastography-based stiffness of the spleen based on post-hoc analysis of MR elastography performed for liver stiffness assessment in a cohort of healthy children aged 7–17.9 years [26].

## Methods

This was a post-hoc analysis of data collected as part of a prospective multicenter cross-sectional study (ClinicalTrials.gov identifier, NCT03235414) [26]. All study activities were Health Insurance Portability and Accountability Act compliant. Data collected under the original study and shared between institutions had been de-identified prior to sharing. No additional ethical approval was required for this post-hoc analysis of de-identified data.

### Study participants

Volunteers aged 7–17.9 years without a known history of liver or spleen disease were recruited at four sites for a research MRI and venous blood draw of approximately 2.5 ml within 24 h of MRI to measure aspartate aminotransferase, alanine aminotransferase, γ-glutamyltransferase, alkaline phosphatase, and total and direct bilirubin. Participating sites were selected based on MRI scanner availability, as follows: Cincinnati Children’s Hospital Medical Center (Philips 1.5 T [T] and 3 T; Philips Healthcare, Best, Netherlands); Children’s Hospital of Philadelphia (General Electric [GE] Healthcare 3 T; GE Healthcare, Milwaukee, WI; and Siemens 3 T; Siemens Healthineers, Erlangen, Germany); Mallinckrodt Institute of Radiology (Siemens 1.5 T); and Massachusetts General Hospital (GE Healthcare 1.5 T). The original study targeted enrollment of 24 volunteers (between February 2018 and October 2019) in each of two age groups at each of two field strengths for a total study recruitment of at least 96 participants (Table 2). Participants were weighed and measured immediately before their MRI examination. Body mass index (BMI) percentiles were calculated using the Centers for Disease Control and Prevention calculator (10). Exclusion criteria included (a) BMI percentile less than 10% or greater than 85%, (b) abnormal blood laboratory value, (c) liver proton density fat fraction (PDFF) greater than 5%, and (d) liver T2* less than 19 ms at 1.5 T or less than 10 ms at 3 T [26].

**Table 2:** MRI scanner and study recruitment details

MRI scanner	Age group (years)	No. of recruited participants
1.5 T GE	7 to 12	8 (4 M and 4F)
	12 to 17.9	8 (4 M and 4F)
3 T GE	7 to 12	8 (4 M and 4F)
	12 to 17.9	11 (6 M and 5F)
1.5 T Philips	7 to 12	8 (4 M and 4F)
	12 to 17.9	8 (5 M and 3F)
3 T Philips	7 to 12	6 (3 M and 3F)
	12 to 17.9	10 (4 M and 6F)
1.5 T Siemens	7 to 12	8 (4 M and 4F)
	12 to 17.9	8 (4 M and 4F)
3 T Siemens	7 to 12	8 (4 M and 4F)
	12 to 17.9	10 (4 M and 6F)

*GE*, General Electric Healthcare; *M*, male; *F*, female; *T*, tesla

The current study reflects post-hoc analysis of data collected from a prospective study of liver MRE for which recruitment was planned to account for MRI scanner vendor, MRI field strength, and age-related difference in normal liver stiffness measured with MR elastography (adapted from the normative liver MRE study) [26]. Data in parentheses are number of male participants and number of female participants

### Research magnetic resonance imaging

**Fig. 1:**
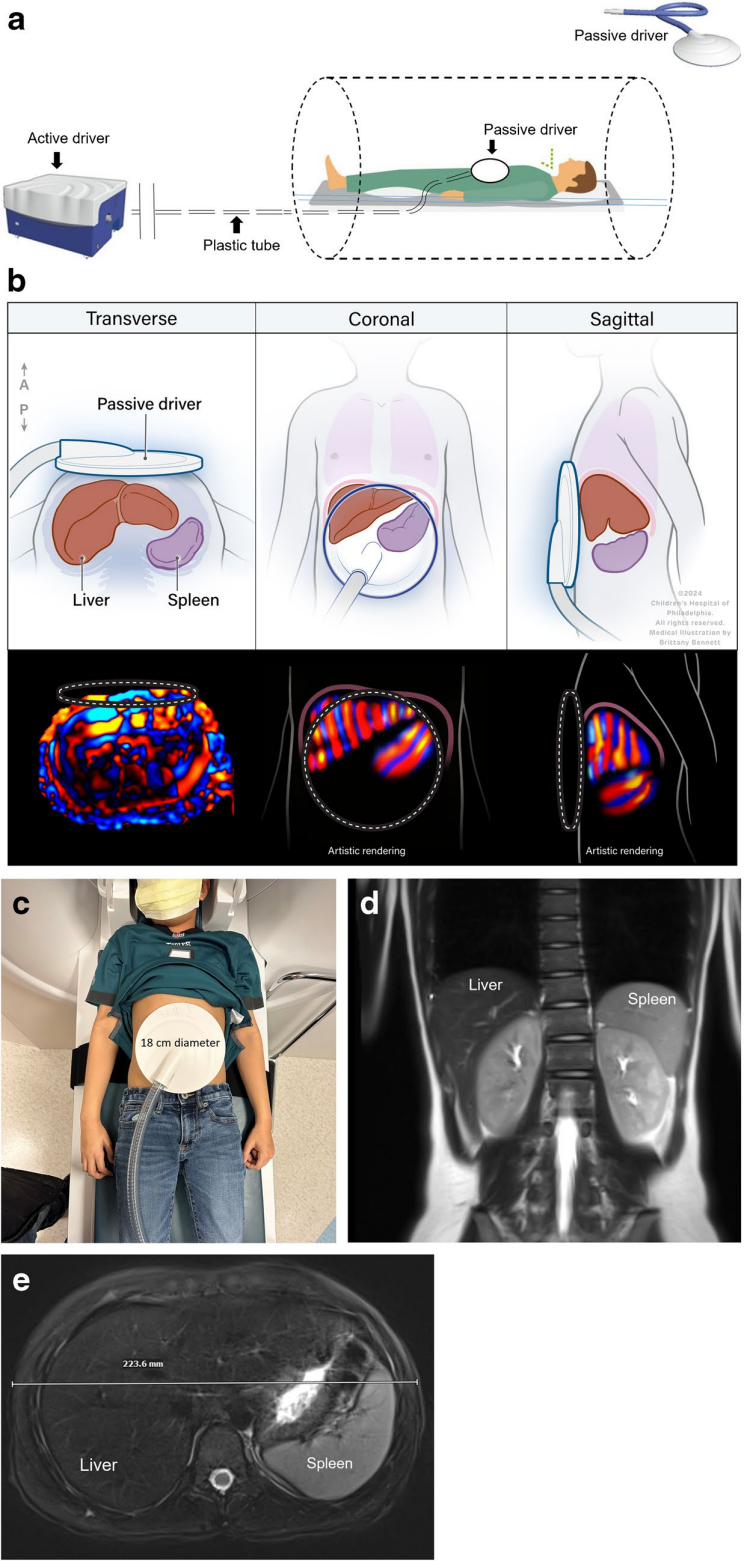
Schematic of patient positioning for magnetic resonance (MR) elastography (**a**), schematic of paddle positioning (**b**), representative photo of an 11-year-old boy positioned for MR elastography study showing that the width of the paddle covers almost the entire abdomen (**c**), coronal T2W slice of a 10-year-old girl showing the anatomic location of liver and spleen (**d**), and axial T2W fat-saturated image (abdomen width = 22.3 cm) (**e**)

Study participants were asked to remain fasting for 4 h before research MRI. Images were acquired with the patient supine in the MRI scanner using an anterior surface array coil. Sequences performed included an axial T2-weighted fast spin-echo sequence for anatomic localization, MR elastography sequence, and liver fat quantification PDFF sequence. Elastography was performed using the manufacturer-recommended MR elastography sequence and an active–passive driver system operated at the manufacturer-recommended frequency of 60 Hz [26]. The passive driver was placed over the right upper quadrant in typical location for measuring liver stiffness (Fig. 1). Because of the size of the passive driver (18 cm), positioning of the paddle over the right upper quadrant while maintaining contact with the abdominal wall in a child results in the passive driver extending over much of the abdomen. For GRE MR elastography, vibration amplitude was adjusted based on participant weight, increasing in 10% increments for each 10 kg of weight from 40% at 49 kg or less to 80% for participants weighing 80–89 kg. All SE-EPI MR elastography examinations were performed with vibration amplitude of 50%. When both were available, the SE-EPI images were used for the current analysis. Following scan acquisition, the scanner generates several images, including a conventional magnitude image, a wave image, and gray-scale stiffness images with and without confidence map overlays.

### Image analysis

**Fig. 2:**
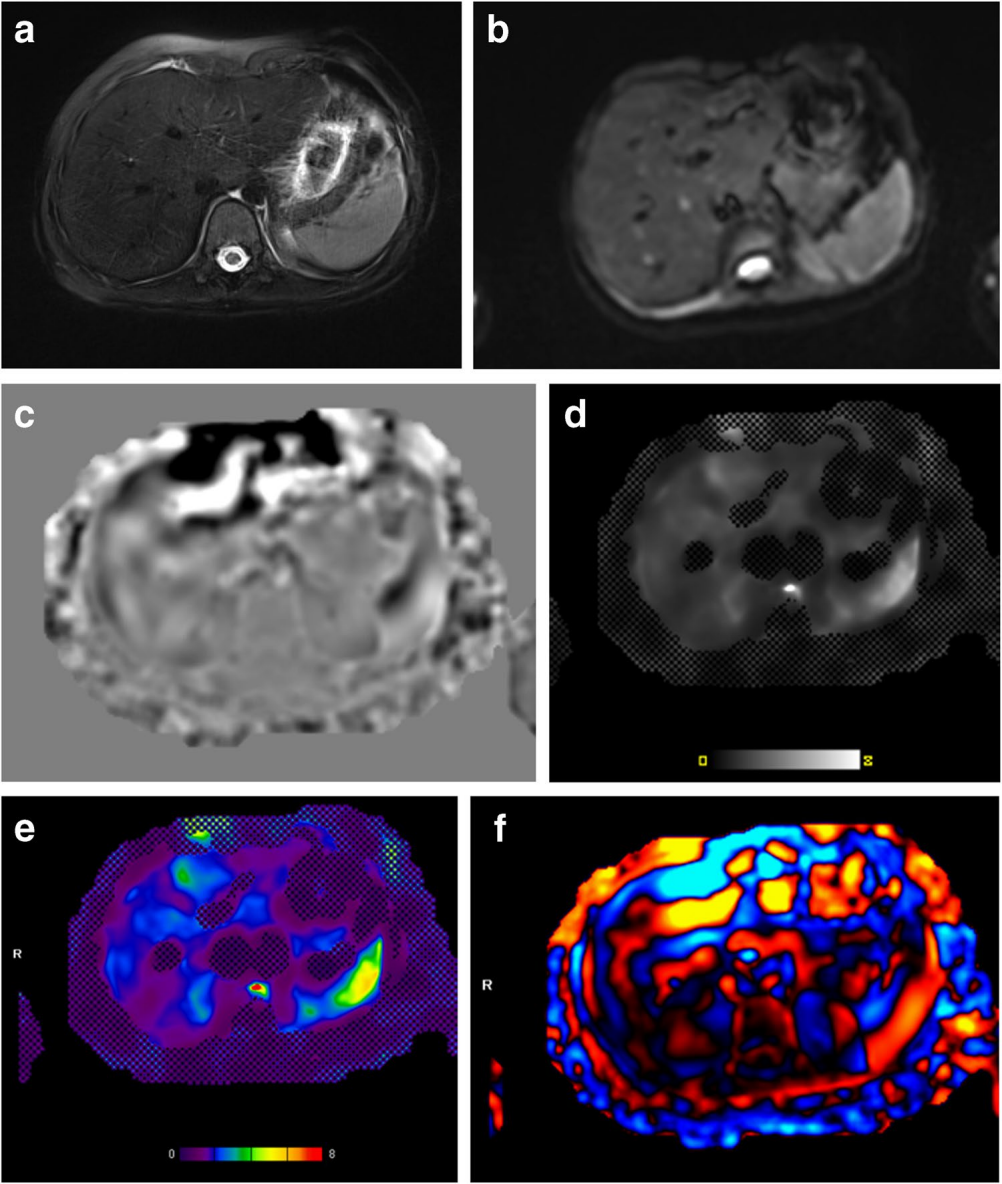
Representative images chosen for quality check for spleen magnetic resonance elastography acquisition of an 8-year-old girl. Axial T2W fat-saturated image (**a**), magnitude image (**b**), gray-scale wave image (**c**), stiffness map with confidence mask (**d**), color stiffness map (**e**), and wave image with red and blue waves seen passing through the spleen (**f**)

For the current study, MR elastography images were reviewed for technical adequacy for splenic measurements by the study principal investigator (A.T.T.), and by an MRI physicist (S.D.S.) independently, both with > 10 years of MR elastography experience. Images were reviewed to confirm that the position of the passive driver was adequate for imaging of both the liver and spleen (Fig. 1). Any discrepancies were mutually resolved. Additionally, images were reviewed for visible propagation of waves through the spleen (Fig. 2). One of the two reviewers also measured abdomen width in the maximum left to right direction using the standard scale function.

**Fig. 3:**
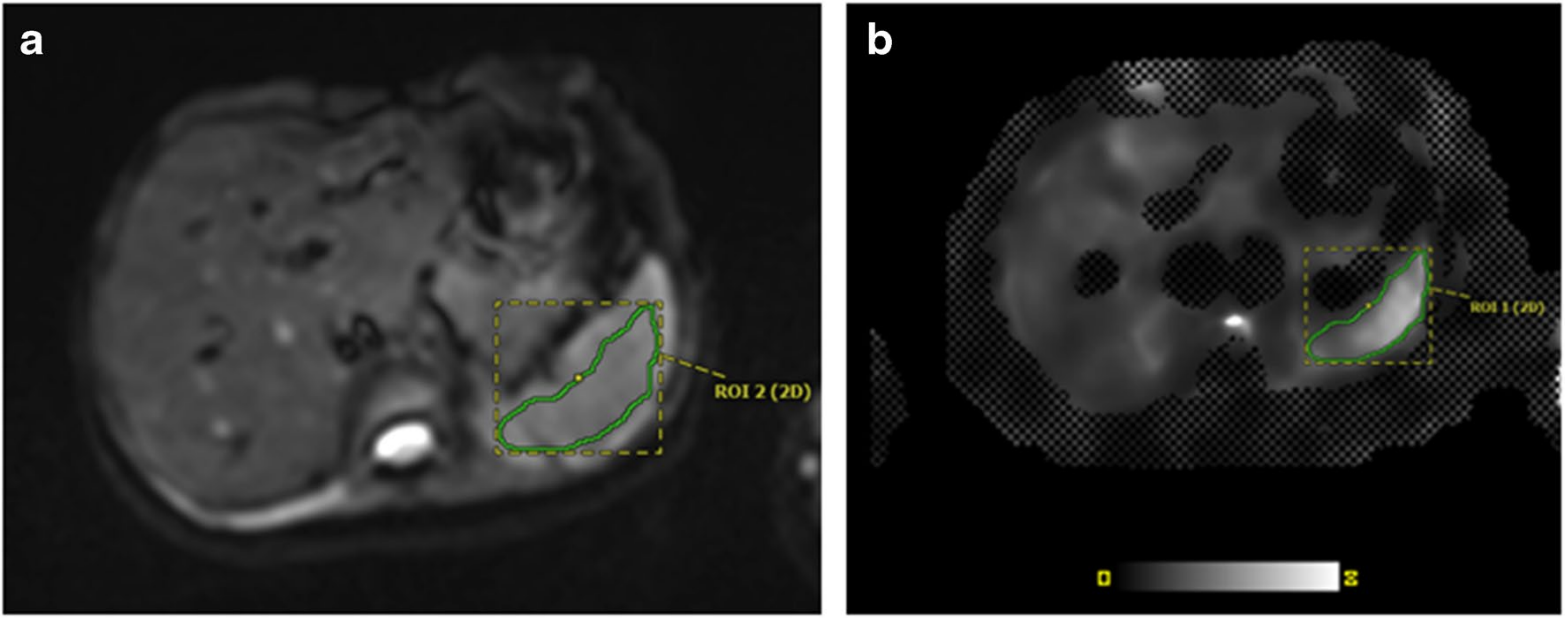
Most preferred region and slice selection for magnetic resonance elastography. Regions of interest drawn on representative magnitude image (**a**) and stiffness map (**b**). Note that slice coverage also includes the spleen

**Fig. 4:**
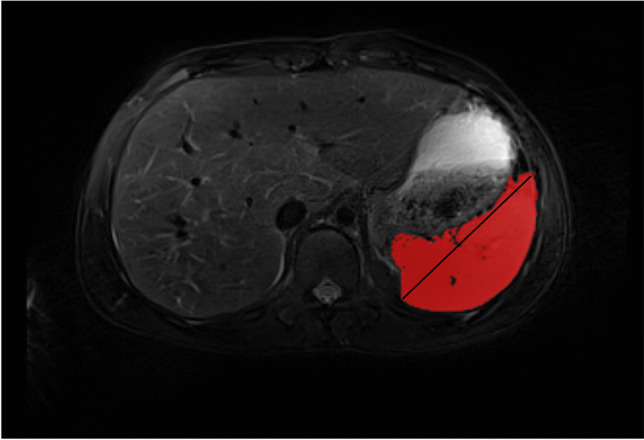
Representative axial image showing spleen length in cranio-caudal dimension (*black line*) and spleen volume region of interest

Two independent reviewers then measured spleen stiffness by manually placing irregular regions of interest (ROI) on two to four stiffness images of the spleen guided by the anatomic imaging (to ensure sampling of the spleen) and the scanner-generated 95% confidence maps (to again ensure sampling of only areas with adequate signal reliability) (Fig. 3). ROIs were drawn on the magnitude image to ensure placement in the spleen and were copy and pasted to the stiffness map. The stiffness values were recorded from the value displayed on the gray-scale stiffness map. The reviewers drawing ROIs were blinded to each other’s measurements and to all participant data. Blinding to scanner manufacturer and field strength was not possible based on image display. Participant spleen stiffness was expressed as a mean of means calculated from the mean stiffness value for each measured region, weighted according to ROI size. Spleen length in the cranio-caudal dimension and spleen volumes were measured by the same observers using parametric MRI software (https://www.parametricmri.com/) (Fig. 4) [21].

### Statistical analysis

Descriptive statistics were expressed as means and standard deviations or medians and interquartile ranges (IQRs) as appropriate for numeric data and frequency and percentages for categorical data. Pearson’s correlation coefficient (*r*) or Spearman’s correlation coefficient (rho) was used to evaluate the correlation between spleen stiffness and continuous variables, as appropriate. To assess relationships between spleen stiffness, length, volume, and age and sex, MRI field strength, scanner vendor, independent sample *t*-test, Mann–Whitney *U*-test, and one-way ANOVA were used. The interobserver agreement between spleen stiffness values for both readers was assessed using a one-way intraclass correlation coefficient (ICC) model. *P* < 0.05 was considered statistically significant. All analyses were performed with STATA (version 18; StataCorp LLC; College Station, TX). Correlation coefficients were interpreted as follows: very strong (*r*/rho = 0.90–1.00), strong (*r*/rho = 0.70–0.89), moderate (*r*/rho = 0.40–0.69), weak (*r*/rho = 0.10–0.39), and negligible (*r*/rho = 0.00–0.10) [27–29].

## Results

From a total of 101 study volunteers, 72 (70.5%) had measurable areas of spleen stiffness and were included in the study analysis. The other 29 (29.5%) participants were excluded because either the wave propagation was deemed inadequate or waves were not clearly visible throughout the spleen.

### Recruited sample

Among the included 72 participants, median age was 12 years (interquartile range [IQR], 9.9–14.9 years) and 34 were female. Mean (± SD) spleen length was 8.7 ± 1.4 cm and mean (± SD) spleen volume was 170 ± 78 ml. Spleen size of participants was observed to be within normal limits [30]. Mean abdomen width (left to right) of the included participants was 25.1 ± 4.1 cm. For the 29 excluded participants, median age was 14.5 years (IQR, 11.5–15.9 years) and 14 were female.

### Spleen stiffness and associations

**Fig. 5:**
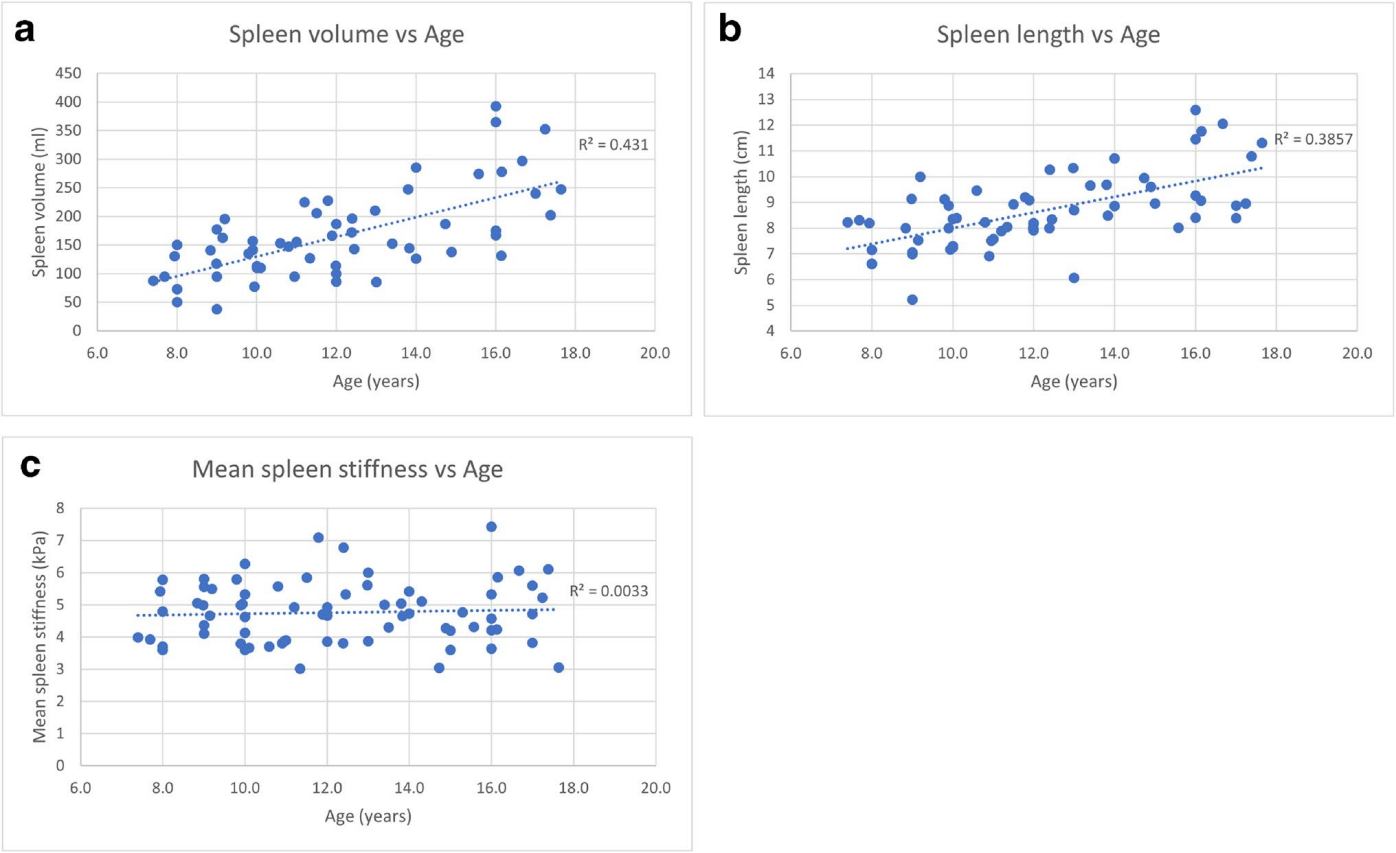
Scatter plot showing the distribution of spleen volume vs. age (**a**), spleen length vs. age (**b**), and mean spleen stiffness vs. age (**c**)

Mean (± SD) spleen stiffness was 4.7 ± 0.9 kPa (IQR, 3.8–5.4 kPa) with 6.1 kPa reflecting the 95th percentile. Male volunteers had slightly higher splenic stiffness and longer spleen length compared to females: 4.9 ± 0.9 vs. 4.3 ± 0.8 kPa (*P* = 0.014), and 9.1 ± 1.8 vs. 8.3 ± 0.8 cm (*P* = 0.034), respectively (Table 3, Fig. 5).

**Table 3:** Summary of patient and spleen characteristics

	Whole sample (*n* = 72)	Males (M) (*n* = 38)	Females (F) (*n* = 34)	*P*-value (M vs. F)
Age (years)	12.5 ± 3.0	12.6 ± 3.1	12.4 ± 2.9	0.684
Spleen stiffness (kPa)	4.7 ± 0.9	4.9 ± 0.9	4.3 ± 0.8	0.014
Spleen length (cm)	8.7 ± 1.4	9.1 ± 1.8	8.3 ± 0.8	0.034
Spleen volume (ml)	170 ± 78	181.7 ± 87.2	153.9 ± 67.3	0.204

**Fig. 6:**
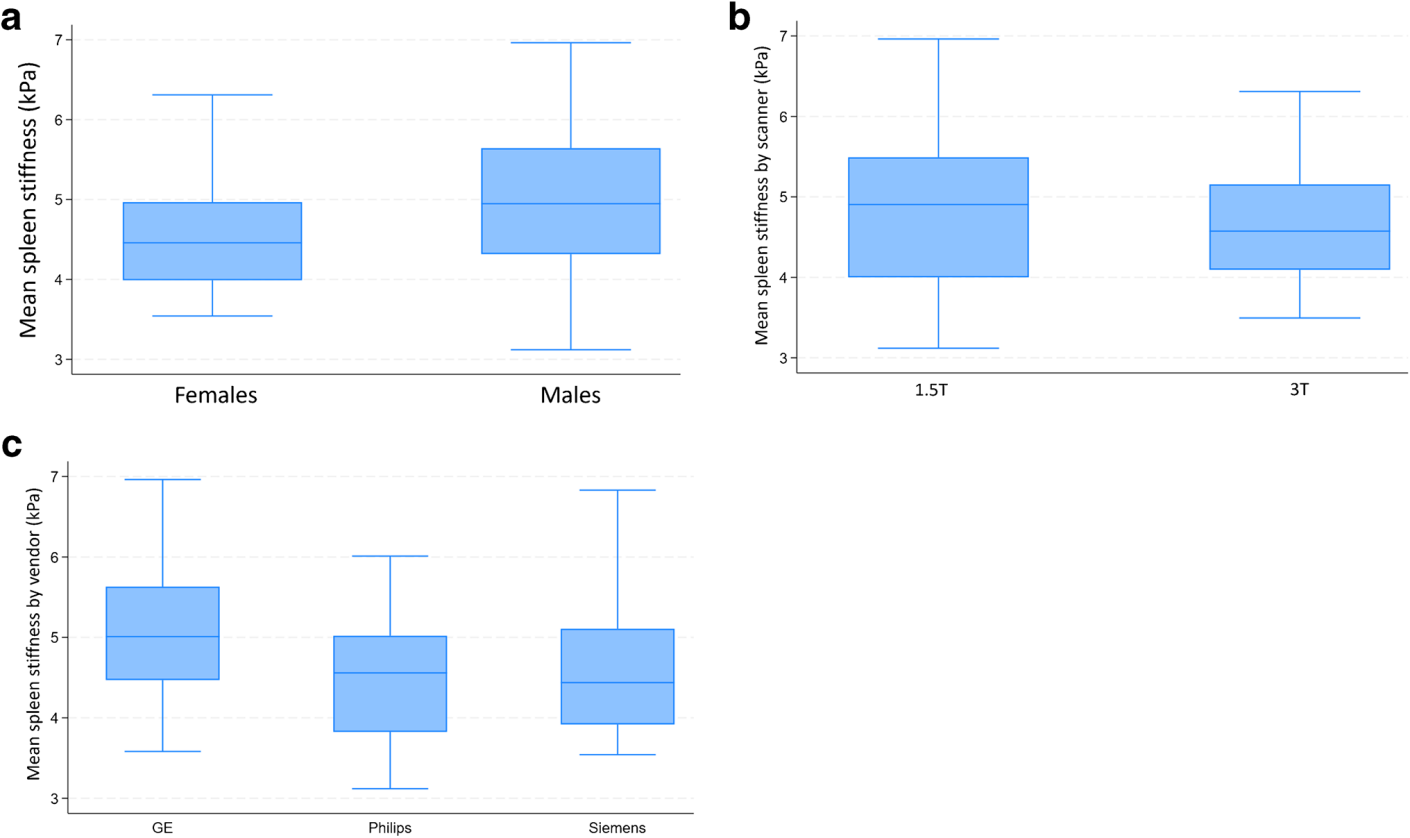
Box and whisker plots of the distribution of measured mean stiffness values (in kilopascals). Mean spleen stiffness values by sex (**a**), mean spleen stiffness values by scanner field strength (**b**), and mean spleen stiffness values by three scanner vendors (**c**)

**Fig. 7:**
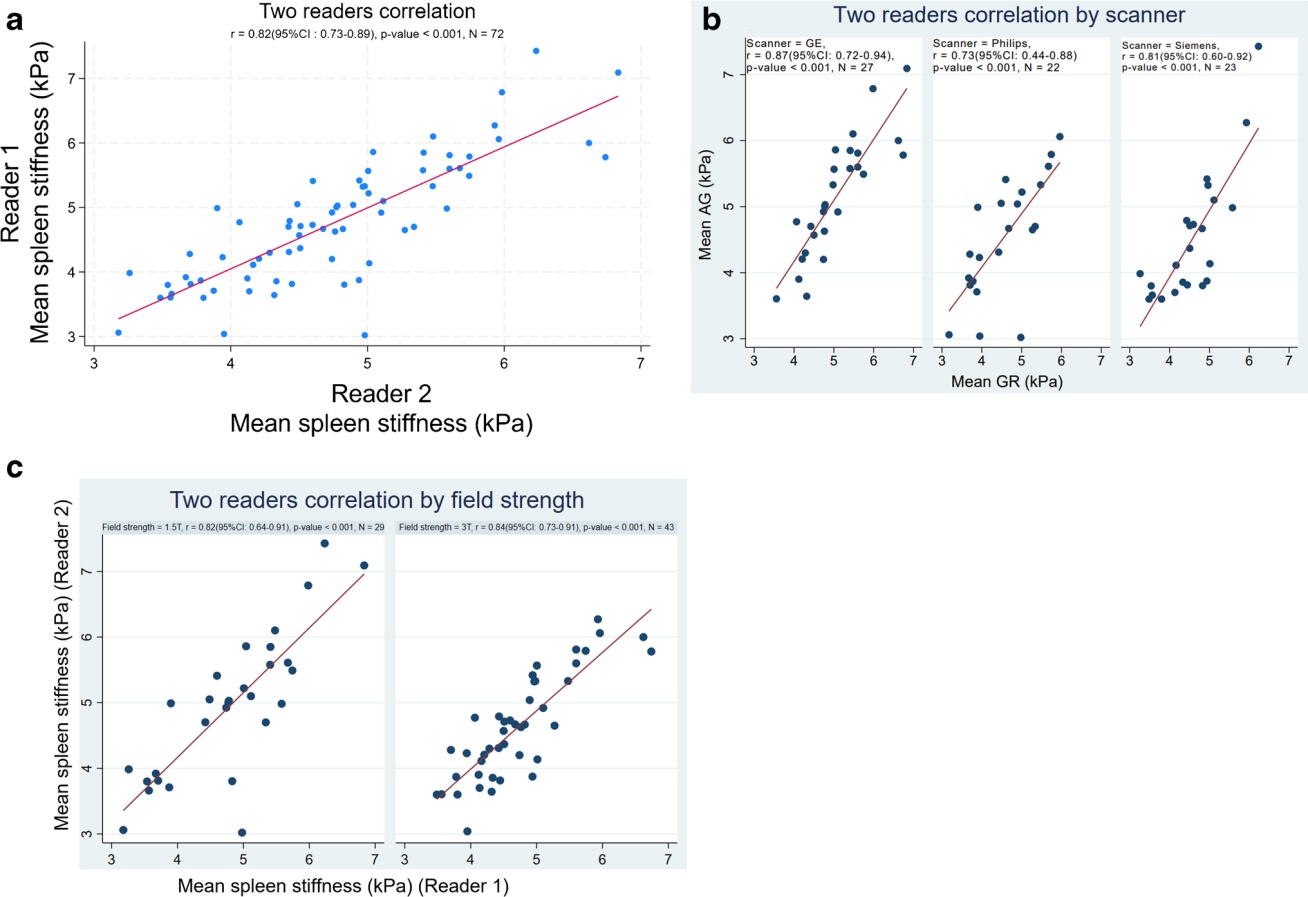
Two readers correlation plot of entire dataset (*r* = 0.82, *P* < 0.001) (**a**), correlation plot of dataset by magnetic resonance imaging (MRI) field strength (*r* = 0.82 for 1.5 T, *n* = 29, *P* < 0.001 and *r* = 0.84 for 3 T, *n* = 43, *P* < 0.001) (**b**), and correlation plot of dataset by three scanner vendors (*r* = 0.87 for GE MRI scanner, *n* = 27, *P* < 0.001; *r* = 0.73 for Philips MRI scanner, *n* = 22, *P* < 0.001; and *r* = 0.81 for Siemens MRI scanner, *n* = 23, *P* < 0.001) (**c**)

There was no significant correlation of spleen stiffness with age (*r* = 0.15 [95%CI, −0.11–0.41; *P* = 0.252]), height (*r* = 0.29 [95%CI, 0.01–0.53; *P* = 0.133]), weight (*r* = 0.19 [95%CI, −0.08–0.46; *P* = 0.140]), BMI% (*r* = 0.23 [95%CI, −0.02–0.47; *P* = 0.078]), or BSA (*r* = 0.17 [95%CI, −0.09–0.43; *P* = 0.18]) (Table 4). However, there was significant correlation between spleen stiffness and spleen length (*r* = 0.37 [95%CI, 0.10–0.59; *P* = 0.007]), and volume (*r* = 0.44 [95%CI, 0.22–0.66; *P* = 0.002]). Spleen size, not spleen stiffness, is impacted by the size of the subject (Fig. 5). No significant difference in spleen stiffness was observed across scanner vendors (*P* = 0.089) or between field strengths (*P* = 0.236) (Fig. 6 and Table 5). Agreement between reviewers was good (*r* = 0.82 [95%CI, 0.73–0.89; *P* < 0.001]) (Fig. 7).

**Table 4:** Univariable correlations between patient variables and spleen stiffness measured with magnetic resonance elastography

Variable	Correlation (95% confidence interval)	*P*-value
Age (years)	0.15 (−0.11–0.41)	0.252
Spleen length (cm)	0.37 (0.10–0.59)	0.007
Spleen volume (ml)	0.44 (0.22–0.66)	0.002
Height	0.29 (0.01–0.53)	0.133
Weight	0.19 (−0.08–0.46)	0.14
BMI	0.33 (0.06–0.56)	0.024
BMI%	0.23 (−0.02–0.47)	0.078
BSA	0.17 (−0.09–0.43)	0.18

*BMI*, body mass index; *BSA*, body surface area

**Table 5:** Bivariate analysis of mean stiffness across MRI field strength and scanner vendor

Variable	MRI field strength			Scanner vendor			
	1.5 T (*n* = 29)	3 T (*n* = 43)	*P*-value	GE (*n* = 27)	Philips (*n* = 22)	Siemens (*n* = 23)	*P*-value
Mean ± SD	4.8 ± 1.2	4.5 ± 0.7	0.236	4.9 ± 0.9	4.3 ± 1.0	4.5 ± 0.7	0.089

*GE*, General Electric Healthcare; *SD*, standard deviation; *T*, tesla

## Discussion

Changes in liver and spleen stiffness measurements are relevant to understanding diffuse liver disease [21, 31] and can serve as a supplemental noninvasive assessment of portal hypertension [31, 32]. Studies have shown splenic stiffness to increase in patients with portal hypertension [33–35]. Based on a post-hoc analysis of previously prospectively collected MR elastography data from a pediatric sample without liver or spleen disease, we report normal spleen stiffness values that may have relevance for defining thresholds for increased splenic stiffness as a marker of disease, particularly portal hypertension. In the included sample, normal mean spleen stiffness was 4.7 ± 0.9 kPa (IQR, 3.8–5.4 kPa) with 6.1 kPa reflecting the 95th percentile. Splenic stiffness was weakly to moderately associated with spleen size and weakly associated with participant BMI but was not associated with scanner manufacturer or scanner field strength.

The normal spleen stiffness value from our pediatric study (4.7 ± 0.9 kPa) is slightly higher than the stiffness value of 3.6 ± 0.3 kPa reported for a group of 12 healthy adult volunteers by Talwalkar et al. [36]. Our results are closer to the 4.25 ± 0.62 kPa mean splenic stiffness reported by Manelli et al. for 16 healthy adult volunteers [37]. The largest study to date of likely normal splenic stiffness was a study by Jhang et al. who reported a mean splenic stiffness of 5.2 ± 1.5 kPa for 115 patients not displaying symptoms of chronic liver disease [25]. This value is comparable to our own findings but was obtained based on a retrospective analysis of medical records.

In our study, we found that pediatric mean spleen stiffness was slightly higher in males than in females. This is concordant with results from the study by Mannelli et al., who also reported spleen stiffness values to be higher for adult men than for women (men, 4.56 ± 0.59 kPa; women, 3.86 ± 0.44 kPa; *P* = 0.017) [37]. The explanation for this observation is uncertain.

Our finding that normal spleen stiffness is not significantly related to MRI scanner manufacturer or field strength is concordant with prior studies of liver stiffness [17, 26]. This suggests that stiffness can be followed across a variety of MR imagers allowing longitudinal monitoring of spleen stiffness as a potential imaging marker of liver disease.

In a previous research study on 49 adult patients, a dual driver configuration was used for simultaneous liver and spleen stiffness estimation [38]. In a development experiment on 11 adult volunteers (mean age, 35.6 years), multiple driver configuration with more than one excitation frequency was tested for assessing the mechanical properties of the liver, spleen, and kidneys simultaneously [39]. The design of passive drivers on both these studies was experimental and insufficiently developed and is not commercially available. There are little data regarding normal spleen stiffness in children aside from what we present in this study. In our sample, a single currently commercially available passive driver excitation seemed sufficient (based on position of the passive driver and visible wave propagation in the spleen) for simultaneous liver and spleen MR elastography in 72 out of 101 subjects. That said, our study is limited by the fact that it was a post-hoc analysis of data collected in a study of liver stiffness, in which the passive driver was placed on the liver and not directly on the spleen. This may have resulted in suboptimal propagation of shear waves to the spleen and our results could be confounded by oblique waves generated by reflection off other body interfaces, such as the diaphragm. Oblique waves will appear to the analysis algorithm as waves of longer wavelength than perpendicular waves. This, in turn, will result in artifactually increased stiffness measurements. Additionally, almost 30% of our subjects had to be excluded because the waves were deemed inadequate. Further work is required to establish whether a single driver location provides repeatable and accurate results for measurements of both spleen and liver stiffness in children.

## Conclusion

Mean 2-D MR elastography-measured spleen stiffness is 4.7 ± 0.9 kPa (IQR, 3.8–5.4 kPa) with 6.1 kPa reflecting the 95th percentile in children aged 7–17.9 years without liver or spleen disease and is not MRI scanner or field strength dependent.

## Data Availability

Data can be made available on a reasonable request.
